# Using the High-Level Based Program Interface to Facilitate the Large Scale Scientific Computing

**DOI:** 10.1155/2014/914514

**Published:** 2014-01-19

**Authors:** Yizi Shang, Ling Shang, Chuanchang Gao, Guiming Lu, Yuntao Ye, Dongdong Jia

**Affiliations:** ^1^State Key Laboratory of Simulation and Regulation of Water Cycle in River Basin, China Institute of Water Resources and Hydropower Research, Beijing 100038, China; ^2^School of Electric Power, North China University of Water Conservancy and Electric Power, Zhengzhou 450045, China; ^3^Dahe Cluture & Finance Group, Nanjing 210007, China; ^4^School of Information Engineering, North China University of Water Conservancy and Electric Power, Zhengzhou 450045, China

## Abstract

This paper is to make further research on facilitating the large-scale scientific computing on the grid and the desktop grid platform. The related issues include the programming method, the overhead of the high-level program interface based middleware, and the data anticipate migration. The block based Gauss Jordan algorithm as a real example of large-scale scientific computing is used to evaluate those issues presented above. The results show that the high-level based program interface makes the complex scientific applications on large-scale scientific platform easier, though a little overhead is unavoidable. Also, the data anticipation migration mechanism can improve the efficiency of the platform which needs to process big data based scientific applications.

## 1. Introduction

The Grid is a generalized network computing system that is supposed to scale to Internet levels and handle data and computation seamlessly. The challenges of designing a grid architecture can be summarized as follows: (1) supporting adaptability, extensibility, and scalability, (2) allowing systems with different administrative policies to interoperate while preserving site autonomy, (3) coallocating resources, (4) supporting quality of service, and (5) meeting computational cost constraints. Many grid projects have been proposed to solve those issues presented above and we will introduce some famous grid projects. The Globus [1–3] project is a multi-institutional research effort that seeks to enable the construction of computational grids providing pervasive, dependable, and consistent access to high performance computational resources, despite geographical distribution of both resources and users. The AppLeS project [4–6] focuses on the design and development of the Grid-enabled high performance schedulers for distributed applications. Legion [7–9] provides the software infrastructure so that a system of heterogeneous, geographically distributed, high performance machines can interact seamlessly. Netsolve [10–12] is a client-agent-server paradigm based network-enabled application server. It is designed to solve computational science problems in a distributed environment. DIET [13] project is to develop a set of tools to build, deploy, and execute computational server daemons. It focuses on the development of the scalable middleware with initial efforts concentrated on distributing the scheduling problem across multiple agents. Ninf allows access to multiple remote computer and database servers. Ninf [14] clients can semitransparently access remote computational resources from languages such as C and FORTRAN. Ninf-G [15] is a reference implementation of the GridRPC [16] API, a proposed GGF standard. Ninf-G aims to support development and execution of Grid applications which will run efficiently on a large-scale computational Grid. SmartGridRPC [17, 18] is an extension of the GridRPC model, which aims to achieve higher performance. SmartGridRPC model has extended the GridRPC model to support collective mapping of a group of tasks by separating the mapping of tasks from their execution. DataCutter [19] is a middleware infrastructure that enables processing of scientific datasets stored in archival storage systems across a wide-area network. DataCutter provides support for subsetting of datasets through multidimensional range queries, and application specific aggregation on scientific datasets stored in an archival storage system. Directed Acyclic Graph Manager (DAGMan) [20, 21] is a metascheduler for the execution of programs. DAGMan submits the programs to Condor in an order represented by a DAG and processes the results. OmniRPC [22] is designed to allow easy development and implementation of parallel scientific applications for distributed and Grid environments. The OmniRPC programming model is very similar to the GridRPC one. It is composed of a client application and various remote computational hosts, which execute the remote procedures. Remote locations can be connected via a local area network or over a wide-area network. The client application can be written in a different language, such as FORTRAN, C, and C++, and the parallel execution in the client can be obtained by using direct thread libraries, such as the POSIX thread, or the OpenMP API. The interface to a remote function is described by the Ninf IDL. In OmniRPC, the remote executions are managed by the use of the remote shell (rsh) for a local distributed environment and by the use of Globus and ssh for a grid environment.

Desktop grid systems can utilize the idle cycles of PC's in Internet environments and/or enterprise environments. The design of the architectural and the organization are the features. We next give an overview of the anatomy of those kinds of systems, summarizing the identifying commonalities and some important differences at the client, application and resource management, and worker levels. On the client level, a user submits an application to the desktop grid, using tools for controlling the application's execution and monitoring its status. On the Application and Resource Management Level, the application is then scheduled on workers and appropriate data will be sent to workers. On the Worker Level, the worker ensures the application's task executes transparently with respect to other user processes on the hosts.

A series of challenges have to be considered when making large-scale computing on the desktop grid system. (1) Volatility (nondedication): since the computing resources of Desktop Grid are mainly from personal computers, it should respect the autonomy of resource providers. That is, volunteers can leave arbitrarily even during the process of the public execution, and they are allowed to execute private execution at any time, thus causing interruption of the public execution. (2) Dynamic environment: resource's owners can configure its preference and can control its facilities in the desktop grid. They can freely join and leave during executions without administrative penalties. Thus, the state of the system (i.e., load, availability, volatility, latency, bandwidth, trust, etc.) is changing over time during the public execution. A scheduler should adapt to such a dynamic environment. (3) Lack of trust: in Desktop Grid, anonymous nodes can participate as a resource provider. Some malicious volunteers tamper with the computation and then return the corrupted results. The desktop grid system should guarantee the correctness of the results. (4) Failure: in desktop grid, volunteers are connected through Internet/low speed network. Therefore, they may experiences more crash and link failures. A robust desktop grid system can tolerate the failures and volatility, once the execution is delayed, blocked, and even lost resulting from that, the volunteers are no longer dedicate to the public service.

XtremWeb [23, 24], an open source middleware to form a global computing platform with a multiuser and multiparallel programming context that intends to distribute applications over dynamic resources. The desktop grid is deployed in a three-tier structure based on the availability of the computing resource, the security policies, and fault-tolerance mechanisms. This architecture gathers the main services in a tree form: Clients, Coordinators, and Workers.

YML-PC [25] is a workflow based private cloud environment for scientific computing. It harnesses dedicated computing resources and volunteer computing resources. To overcome the shortage from the volatility of the desktop grid environment, a trust model [26] is introduced in the study. Experiments from paper [25] testify that data dependent based DAG tasks can be dealt with in desktop grid environment.

This paper aims to make further research on issues with the large-scale computing platform. As it is well known to us all, programing for the large-scale scientific platform is not an easy task, especially for those programs where data dependence between operations exist. Each grid middleware has its programming environment, and those environments are diverse. It is hard for non-IT-professional scientists to master all. To overcome this kind of shortage, a higher level program interface based middlewares has been proposed. Such as YML, DAGMan, YML-PC. While, it is unavoidable to add overhead using those high-level based program interface based middlewares. The first goal of this paper is to evaluate complexity between different program interfaces and overhead generated by additional process. The second goal is to evaluate the influence from different data transfer mechanisms. Generally speaking, data dependence based operations are hard to deal with in grid and desktop grid environments for the uncertainties of task finishing time and data transfer time. In worst case scenarios, the operation perhaps can never be finished. But an appropriate data transfer model can help to solve the problems presented above. So, the data transfer model and schedule mechanism are very important issues for large-scale platform. In this paper, we will focus on the data transfer model on condition that the schedule mechanism is perfectly matched with the platform.

The remainder of the paper is organized as follows. The introduction of block based Gauss Jordan (BbGJ) algorithm will be made and the analysis of data dependence in BbGJ will be discussed in Section 2.  Section 3 will show how the high-level based program interface makes the large-scale scientific platform use easy, and the way to realize data anticipation migration on the platform is given in Section 5.  Section 6 is the conclusion and future works.

## 2. Block Based Gauss Jordan Algorithm

The sequential BbGJ algorithm [27–29] is a classical linear algebra method to get the inversion of a large-scale matrix. The algorithm can be described as follows. Let *A* and *B* be two dense matrices of dimension *N*, and let *B* be the inverse of *A*, that is, *AB* = *BA* = *I*. Let *A* and *B* be partitioned into a matrix of *q* × *q* blocks of dimension *n* which *n* = *N*/*q*.

**Figure 1:**
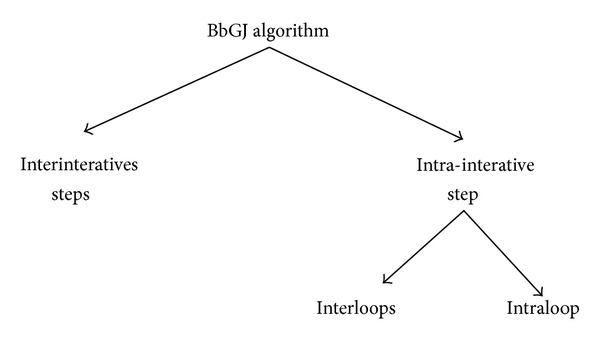
Analysis of parallelism on BbGJ algorithm.

The parallelization consists of exploiting two kinds of parallelism: the intersteps based parallelism and the intrastep based parallelism. The intrastep parallelism aims at exploiting the parallelism involved in each of the five loops. It falls into two categories: the interloops parallelism and the intraloop parallelism. See details in Figure 1.

**Figure 2:**
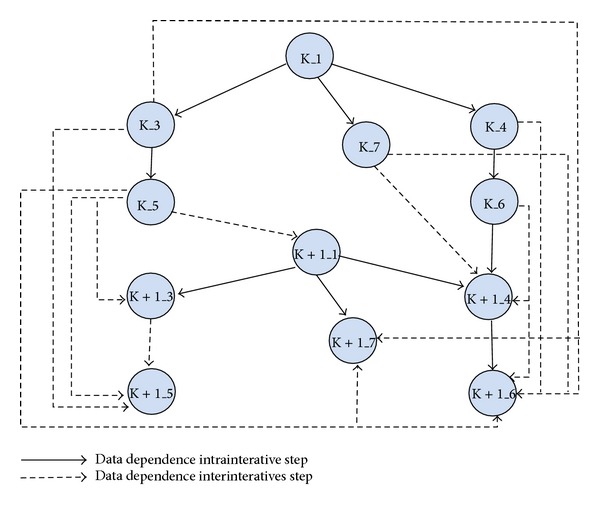
Data dependence between operations.

We summarize all the data dependence existing in BbGJ algorithm using Figure 2.

**Figure 3:**
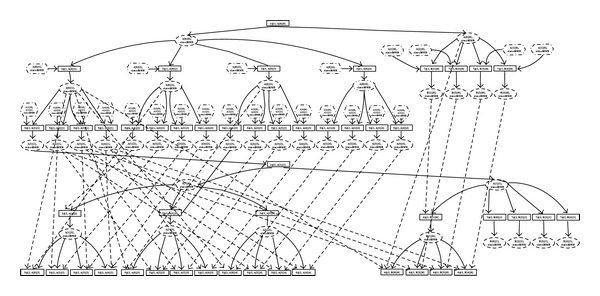
Data transfer model in BbGJ algorithm.

Data transfer model in BbGJ algorithm can be described as follows. See details in Figure 3.

## 3. High-Level Based Program Interface for Large-Scale Scientific Platform

There are lots of high-level program interface based middlewares, and for the study we choose YML as the representative. YML [30–32] is a framework which provides high-level program interface. The aim of YML is to define an abstraction for middlewares, hiding differences among them, and using this abstraction to remain portable over multiple middlewares. Thus, YML can provide less time to solution in scientific computing areas for end users. The user can easily develop a complex parallel application which may execute on multiple middlewares transparently. The framework is divided into three parts: “end-users interface,” “frontend,” and “backend.”

Programming with the OmniRPC and the XtremWeb requires users to know about their APIs and the computing environments they would like to use. That is to say, the users have to deal with something before gridificating their application. Firstly, users must know how to adapt their application to Grid/Desktop Grid environment through APIs OmniRPC/XtremWeb provided. Secondly, users also must know more information about platforms. They need to know the status of computing resources and how to allocate tasks to related computing resources. The process of using XtremWeb/OmniRPC is complex for end users. Last, but not the least, it is hard to reuse the developed code.

**Figure 4:**
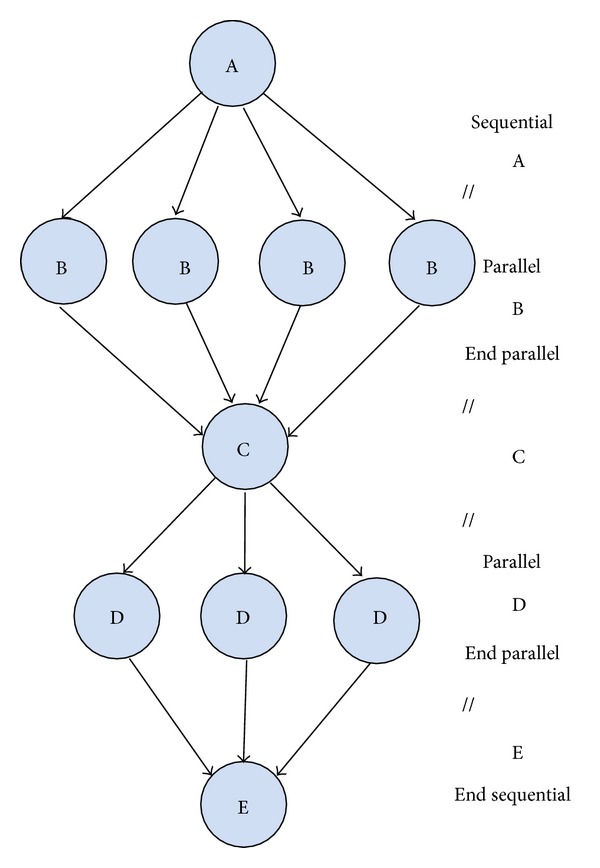
Pseudocode based program model for large-scale scientific computing.

The YML provides end users a higher level programming interface which is a pseudocode. See it in Figure 4. The advantage of the YML is that it succeeds in separating “operation functions” from “control flow.” The “operation functions” (e.g., “operation 3” in BbGJ algorithm) can be developed by a third party or invoking related functions from common libraries. At the same time, this separation makes those “operation functions” very easily reused. End users need not have a specific knowledge of programing those “operation functions.” The interface of the YML is just to describe the “control flow” of application program and it is independent of the program language and the underlying execution environments. So, if users know more details about the application, it is very easy for end users to program with the YML since it provides a vivid interface description.

The YML is intended to provide end users with a very user friendly interface. By the use of YML, the programming becomes easy as compared with OmniRPC or XtremWeb. A user friendly interface can save lots of costs in time (high-level interface makes programing easier) and money (reused component, once develop, and many times use) for end users. Here, we should point out that the YML is developed based on OmniRPC/XtremWeb and some overheads are added to the platform. In the next section, we will discuss the overhead by adopting the YML framework.

From the description in the last section, we can know about two points as follows.

- YML supports the separation of “control flow” and “executable functions” and it helps end users just focus on parallel algorithm itself without considering how to adapt their application to detail executable environments. Based on xml based description programming language, YML provides a high-level programming interface which is very easy to use.
- YML is based on some middleware. YML compiler will generate a schedule table through parse pseudocode based application program developed using YvetteML. Then, YML scheduler will allocate appropriate tasks to available YML workers. YML worker will put available tasks to related computing resources according to its local scheduler. Now, it can support two middlewares: XtremWeb and OmniRPC.

## 4. Evaluation on the Overhead

From the description above, we can know that it is reasonable for the YML to have some overhead. The overhead mainly comes from two aspects as follows.

- YML need to invoke related “implementation components” from YML server. While, OmniRPC invoke their related “implementation functions” from local server.
- YML server has to deal with “scheduler table” when each event happens. Even when the scheduler table is very big, the overhead will become larger.

The following experiments will testify that the high-level program interface based middleware can be a good solution for large-scale scientific computing though a little overhead is unavoidable.

Experiment environment: 100 nodes used in grid environment. Experiment data: we change the block-count of submatrix from 2∗2,  3∗3,  4∗4,  5∗5,  6∗6,  7∗7, and  8∗8. We also change the block-size of submatrix from 500∗500,  1000∗1000 to 1500∗1500.

**Figure 5:**
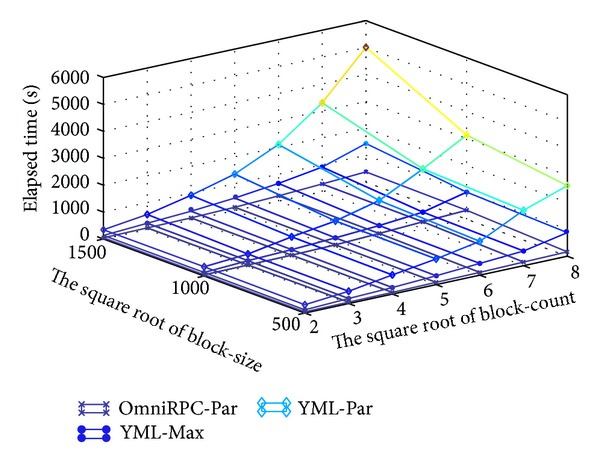
Overhead of YML on OmniRPC.

From Figure 5, we can find the overhead of YML on OmniRPC through comparing execution time of Par-par BbGJ algorithm on YML and that on OmniRPC. The results show that overhead is unavoidable. But its overhead is neglectful. The performance of Max-par BbGJ algorithm on YML is very close to that of Par-par BbGJ algorithm on OmniRPC. At the same time, we know programming Max-par OmniRPC with YML is very easy and it is more difficult to program Max-par algorithm on OmniRPC. The reason is that there are too much complex control events which are used to deal with concurrency of application program. The complex control events make programming using OmniRPC become more difficult. In other words, YML can reduce the time to solution of running a new algorithm through its easy-to-use interface and it also can reduce cost to solution through components reuse. So, YML can be a good choice for end users to facilitate large-scale scientific computing.

Through experiments, we have testified that YML which is the high-level program interface based middleware can be a good choice of achieving less time to solution. Now, we would like to show another feature which is the portability between different kinds of platforms. This feature is unique and few middleware can posse this kind of capability. Using YML based program can run on the grid platform and the desktop grid platform without any change. It is an appealing point for those scientific researchers.

Next, we will devise experiments based on XtremWeb using Par-par BbGJ algorithm on the desktop grid platform.

Experiment data: we change the block-count of submatrix from 2∗2,  4∗4,  6∗6 to  8∗8. We set the block-size of submatrix as 1500∗1500. The bandwidth between site 1 and site 3 is 1 GB/s. The detailed environment can be described using Table 1.

Through running the program for XtremWeb based desktop grid platform, we get the results presented in Table 2. Here, we may not be able to compare the middleware XtremWeb with the middleware of OmniRPC, for those two middleware belong to two different kinds of middlewares adapting to different running environments. However, high-level program interface based middleware may be a good way to adapt the program to suit different environments (Grid environment or Desktop Grid environment). Using this kind of middleware, the programs can be migrated between different environments without any change. With the success of Seti@home, more and more scientific computing will try to use volunteer computing resources for their lower costs and huge processing power.

Through the experiments, we also know that the YML has an acceptable overhead, furthermore, it can help to reduce costs on time and money for users through high-level programming interface and reusable components.

In summary, the YML can be a good choice for large-scale computing and the outstanding advantages are given as follows.

- YML has an acceptable overhead and it can help users to reduce time to solution through its high-level program interface.
- The separation of “implementation component” from “control flow” makes the developed code be reused very easily, which help users to reduce cost-to-solution through components reuse.
- YML support program migration between different executable environments (e.g., Grid, Desktop Grid) without changing the developed code. This is very special and an important character for YML and few other middleware can do this.

## 5. Data Anticipation Migration Mechanism

**Figure 6:**
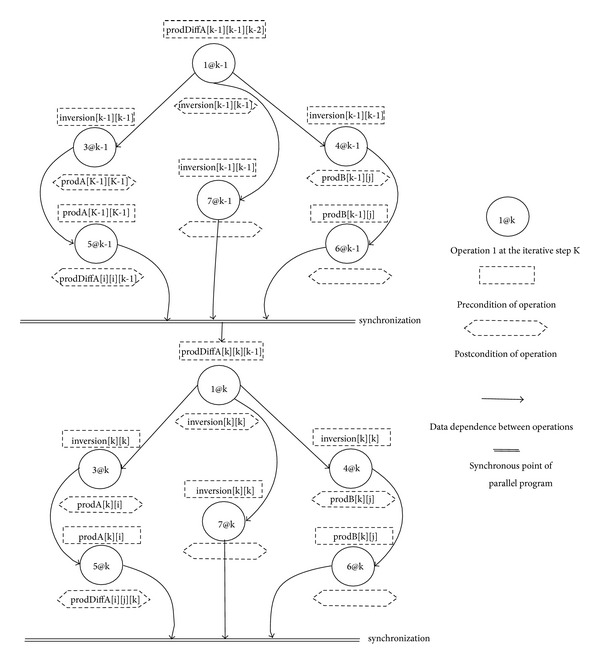
Data transfer model between operations.

As well known to us all, the communication time is a key issue in making large-scale computing. The data transfer model of middleware is traditionally built based on the data server-worker architecture, see Figure 6. So, improving data transfer model can be a good solution to improve the performance of platform, especially in low speed network based environment. And we have done much effort to improve it, and in this section, two experiments are employed to show the improvement.

The method is that we will generate as little communication as possible during the process of program execution. If a data migration is needed in the program, we will not transfer the related data from the data server to the target computer. Otherwise, we will put related operations on appropriate computing nodes. Therefore, less communication is created.

The facilities used in the experiment are described in Table 1. And we will run Max-par BbGJ algorithm on low speed network based PCs. See Table 3.

Another experiment environment is based on high speed network based platform. We also make the experiment using Max-par BbGJ algorithm. See Table 4.

From the experiments, we conclude that with data transfer model, the performance of the platform can improve almost 50% under the desktop grid environment and about 40% under the grid environment, respectively. What we want to point out is that in desktop grid environment, the data transfer model can help to save more time.

## 6. Conclusion

From the presentation above, we testify that high-level program interface based middleware can be a very good solution for end users to make large-scale scientific computing. The performance of the data transfer model affects the overall efficiency of the platform, and a better data transfer mechanism can greatly improve the efficiency of large-scale platform.

The experiment results show that though a little overhead is unavoidable, the high-level program interface based middleware still can be a good solution to reduce developing time and complexity of scientific application. Besides, the codes with high-level program interface based middleware can be migrated between the grid environment and the desktop grid environment without any changes.

In general, we conclude that the high-level program interface based middleware makes end-users program easier whatever the running environments are. The “separation” of “control flow” and “implementation component” enables the end users to focus on the application itself without knowing the programing languages. The “separation” feature makes the developed code reused very easily.

## Figures and Tables

**Table 1:** Computing resources used in platform.

Site	Nodes	Bandwidth	CPU/memory
Site 1	16	100 MB/s	Inter, 2.66 GHz/512 M
Site 2	64	100 MB/s	AMD, 1.8 GHz/512 M

**Table 2:** Overhead of YML on XtremWeb.

	XtremWeb	YML + XtremWeb	Overhead
1500∗2	608	727.88	119.88
1500∗4	3675	3943.7	268.7
1500∗6	8943.67	9704.47	760.8
1500∗8	17633.4	19736.2	2102.8

**Table 3:** Block-count is 5∗5 on PolyTech Lille platform.

Algorithm	Block-size				
	100∗100	200∗200	300∗300	400∗400	500∗500
With DTM	123.25	198.45	251.46	368.98	563.87
No DTM	214.87	328.87	498.13	760.33	1073.60
Gain	42%	40%	49.5%	51.5%	47.5%

**Table 4:** Block-count is 5∗5 on Grid5000 platform.

Algorithm	Block-size				
	100∗100	200∗200	300∗300	400∗400	500∗500
With DTM	6.27	9.87	12.31	37.74	52.33
No DTM	4.19	5.98	7.48	21.04	32.56
Gain	33%	39.4%	39.2%	43.2%	37.8%

## References

[1] Foster I, Kesselman C The Globus project: a status report.

[2] Foster I, Kesselman C (1997). Globus: a metacomputing infrastructure toolkit. *International Journal of High Performance Computing Applications*.

[3] Foster I Globus toolkit version 4: software for service-oriented systems. *IFIP International Conference on Network and Parallel Computing*.

[4] Berman F, Wolski R The AppLeS project: a status report.

[5] Casanova H, Obertelli G, Berman F, Wolski R The AppLeS parameter sweep template: user-level middleware for the Grid.

[6] Berman F, Wolski R, Casanova H (2003). Adaptive computing on the grid using AppLeS. *IEEE Transactions on Parallel and Distributed Systems*.

[7] Wells S (2003). *Legion 1. 8 Basic User Manual*.

[8] Chapin SJ, Katramatos D, Karpovich JF, Grimshaw AS (1998). Resource management in Legion. *University of Virginia Technical Report*.

[9] Natrajan A, Humphrey MA, Grimshaw AS (2002). The legion support for advanced parameter-space studies on a grid. *Future Generation Computer Systems*.

[10] Arnold D, Agrawal S, Blackford S (2001). Users' guide to NetSolve V1. 4. *Computer Science Department Technical Report*.

[11] Casanova H, Dongarra J (1997). Netsolve: a network-enabled server for solving computational science problems. *International Journal of High Performance Computing Applications*.

[12] YarKhan A, Dongarra J, Seymour K Grid-based problem solving environments: implications for development and deployment of numerical software.

[13] Caron E, Desprez F (2006). Diet: a scalable toolbox to build network enabled servers on the grid. *International Journal of High Performance Computing Applications*.

[14] Nakada H, Sato M, Sekiguchi S (1999). Design and implementations of Ninf: towards a global computing infrastructure. *Future Generation Computer Systems*.

[15] Tanaka Y, Nakada H, Sekiguchi S, Suzumura T, Matsuoka S (2003). Ninf-G: a reference implementation of RPC-based programming middleware for Grid computing. *Journal of Grid Computing*.

[16] Tanaka Y, Takemiya H, Nakada H, Sekiguchi S Design, implementation and performance evaluation of GridRPC programming middleware for a large-scale computational Grid.

[17] Brady T, Guidolin M, Lastovetsky A Experiments with smartgridsolve: achieving higher performance by improving the gridRPC model.

[18] Guidolin M, Lastovetsky A ADL: an algorithm definition language for SmartGridSolve.

[19] Beynon MD, Ferreira R, Kurc T, Sussman A, Saltz J DataCutter: middleware for filtering very large scientific datasets on archival storage systems.

[20] Neubauer F, Hoheisel A, Geiler J (2006). Workflow-based Grid applications. *Future Generation Computer Systems*.

[21] Qin J, Fahringer T Advanced data flow support for scientific Grid workflow Applications.

[22] Sato M, Boku T, Takahashi D OmniRPC: a Grid RPC system for parallel programming in cluster and Grid environment.

[23] Cappello F, Djilali S, Fedak G (2005). Computing on large-scale distributed systems: XtremWeb architecture, programming models, security, tests and convergence with grid. *Future Generation Computer Systems*.

[24] Fedak G, Germain C, Neri V, Cappello F XtremWeb: a generic global computing system.

[25] Shang L, Petiton S, Emad N, Yang X YML-PC: a reference architecture based on workflow for building scientific private clouds. *Cloud Computing*.

[26] Shang L, Wang Z, Zhou X, Huang X, Cheng Y Tm-dg: a trust model based on computer users' daily behavior for desktop grid platform.

[27] Shang L, Petiton S, Hugues M A new parallel paradigm for block-based Gauss-Jordan algorithm.

[28] Shang LS, Hugues M, Petiton SG (2010). A fine-grained task based parallel programming paradigm of Gauss-Jordan algorithm. *Journal of Computers*.

[29] Shang Y, Lu G, Shang L, Wang G (2011). Parallel processing on block-based Gauss-Jordan algorithm for desktop grid. *Computer Science and Information Systems*.

[30] Delannoy O, Emad N, Petiton S Workflow global computing with YML.

[31] Delannoy O, Petiton S A peer to peer computing framework: design and performance evaluation of YML.

[32] Delannoy O (2008). *YML: a scientific workflow for high performance computing [Ph.D. thesis]*.

